# Missouri Valley Veterinary Association

**Published:** 1900-07

**Authors:** Joseph W. Parker

**Affiliations:** Secretary


S. STEWART,

Secretary.

### MISSOURI VALLEY VETERINARY ASSOCIATION.

The twenty-fourth regular meeting of the Missouri Valley Veterinary Association convened at the Y. M. C. A. building, St. Joseph, Mo., on June 25, 1900; regular officers, Dr. John Forbes, President, and Dr. John B. Wright, Secretary, in their respective places, and with the following members, visiting veterinarians and others present: Dr. R. C. Moore, Dr. S. Stewart, Dr. Charles A. Monney, Dr. H. C. Babcock and Dr. J. W. Parker, of Kansas City; Dr. H. V. Goode, Dr. E. J. Netherton, Dr. H. J. Washburn, Dr. J. A. Sloan, Dr. J. E. Blackwell, Dr. Charles E. Steel, Dr. Ripley and Dr. Lockwood, of St. Joseph, Mo.; Dr. G. R. Conrad, of Sabetha, Kans., Dr. John Ernst, of Leavenworth, Kan., and Dr. L. D. Brown, of Hamilton, Mo.

During the afternoon an interesting surgical clinic had been held at Dr. E. J. Netherton's Veterinary Hospital, Drs. Steel and Netherton performing a variety of operations in a creditable manner.

The Board of Censors reported favorably on applications by Dr. Charles E. Steel and Dr. Joseph W. Parker for membership, and they were accordingly elected active members.

The following officers were chosen for the ensuing year: Dr. John Forbes, re-elected President; Dr. L. D. Brown, First Vice-President; Dr. John Ernst, Second Vice-President; Dr. J. W. Parker, Secretary and Treasurer; Dr. J. A. Sloan, Dr. E. J. Netherton, Dr. C. E. Steel, Dr. J. B. Wright and Dr. R. G. Conrad, Board of Censors.

The retiring Secretary-Treasurer, Dr. J. B. Wright, submitted a report of the business and condition of the Association. The report shows regular meetings well attended, valuable papers contributed, and that the Association is in a thriving condition.

The literary programme of the evening was as follows: A paper on "Education of Lawmakers," by Dr. J. W. Parker, being a plea for united action in the present campaign and with the legislative bodies of Missouri, Iowa and Kansas, for adequate veterinary laws. The discussion was led by Dr. S. Stewart, who ably supplemented the thoughts advanced by the essayist. General discussion participated in by nearly all present, and at the close of the discussion Dr. E. J. Netherton offered a motion that the President appoint a committee of five to co-operate with like committees of the Veterinary Associations of Missouri, Iowa and Kansas, with a view of securing better veterinary legislation. Being duly seconded, it was so voted unanimously. The President appointed Drs. Stewart, Parker, Netherton, Moore and Goode.

Dr. J. A. Sloan presented a well-prepared paper on "Healing of External Wounds," dealing especially with the processes involved. Discussion led by Dr. H. J. Washburn and participated in by Drs. Moore, Stewart, Steel, Netherton, Parker and Lockwood.

Dr. R. C. Moore read an instructive paper on "Defective Eyelids," restricting it to congenital malformations. The discussion, which was led by Dr. C. E. Steel, brought out many interesting and instructive points. Elsewhere in the JOURNAL will be found the papers presented.

The next meeting of the Association will be held at the Kansas City Veterinary College, 1406 Holmes Street, Kansas City, Mo., September 17, 1900, and a sufficient number of papers have already been promised to guarantee an interesting programme.

JOSEPH W. PARKER,
Secretary.

